# 3-(3-Chloro­phen­yl)-*N*-phenyl­oxirane-2-carboxamide

**DOI:** 10.1107/S1600536809045553

**Published:** 2009-11-04

**Authors:** Long He, Lian-Mei Chen

**Affiliations:** a College of Chemistry and Chemical Engineering, China West Normal University, Nanchong 637002, People’s Republic of China

## Abstract

There are two independent mol­ecules in the asymmetric unit of the title compound, C_15_H_12_ClN_2_O_2_. In each mol­ecule, the two benzene rings adopt a *cis* configuration with respect to the ep­oxy ring. The dihedral angles between the ep­oxy ring and chloro­phenyl rings are essentially identical in the two mol­ecules [62.50 (9) and 62.67 (9)°]. Inter­molecualar N—H⋯O and C—H⋯O hydrogen bonding is present in the crystal structure.

## Related literature

For the use of epoxide-containing compounds as building blocks in the synthesis of a wide range of polyfunctional compounds, see: Imashiro & Seki (2004 ▶); Porter & Skidmore (2000 ▶); Shing *et al.* (2006 ▶); Zhu & Espenson (1995 ▶). For a related structure, see: He (2009 ▶).
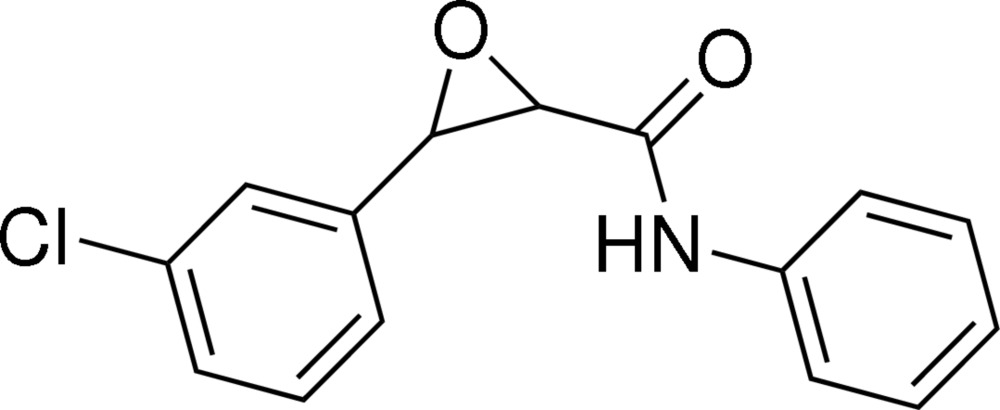


## Experimental

### 

#### Crystal data

C_15_H_12_ClNO_2_
*M* *_r_* = 273.71
Monoclinic, 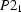

*a* = 5.4480 (1) Å
*b* = 11.1481 (2) Å
*c* = 21.3152 (4) Å
β = 94.472 (2)°
*V* = 1290.63 (4) Å^3^
*Z* = 4
Cu *K*α radiation
μ = 2.60 mm^−1^
*T* = 295 K
0.40 × 0.30 × 0.30 mm

#### Data collection

Oxford Diffraction Gemini S Ultra diffractometer
Absorption correction: multi-scan (*CrysAlis Pro*; Oxford Diffraction, 2009 ▶) *T*_min_ = 0.423, *T*_max_ = 0.510
19148 measured reflections
4775 independent reflections
4453 reflections with *I* > 2σ(*I*)
*R*_int_ = 0.030

#### Refinement

*R*[*F* ^2^ > 2σ(*F* ^2^)] = 0.028
*wR*(*F* ^2^) = 0.072
*S* = 1.00
4775 reflections
351 parameters
3 restraints
H atoms treated by a mixture of independent and constrained refinement
Δρ_max_ = 0.16 e Å^−3^
Δρ_min_ = −0.32 e Å^−3^
Absolute structure: Flack (1983 ▶), 2223 Friedel pairs
Flack parameter: 0.000 (9)

### 

Data collection: *CrysAlis Pro* (Oxford Diffraction, 2009 ▶); cell refinement: *CrysAlis Pro*; data reduction: *CrysAlis Pro*; program(s) used to solve structure: *SHELXS97* (Sheldrick, 2008 ▶); program(s) used to refine structure: *SHELXL97* (Sheldrick, 2008 ▶); molecular graphics: *ORTEP-3* (Farrugia, 1997 ▶); software used to prepare material for publication: *SHELXL97*.

## Supplementary Material

Crystal structure: contains datablocks global, I. DOI: 10.1107/S1600536809045553/xu2659sup1.cif

Structure factors: contains datablocks I. DOI: 10.1107/S1600536809045553/xu2659Isup2.hkl

Additional supplementary materials:  crystallographic information; 3D view; checkCIF report

## Figures and Tables

**Table 1:** Hydrogen-bond geometry (Å, °)

*D*—H⋯*A*	*D*—H	H⋯*A*	*D*⋯*A*	*D*—H⋯*A*
N1—H1⋯O1^i^	0.899 (14)	2.411 (11)	3.2287 (15)	151.5
N2—H16⋯O4^i^	0.887 (13)	2.404 (11)	3.2357 (16)	156.3
C4—H4⋯O1^i^	0.93	2.49	3.388 (2)	162
C15—H15⋯O1^i^	0.93	2.58	3.259 (2)	130
C19—H19⋯O4^i^	0.93	2.59	3.5087 (19)	168

Symmetry code: (i) 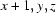.

**Fig. 1.**
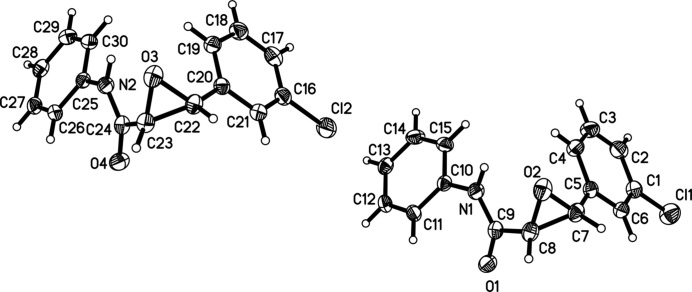
The molecular structure of (I) with 30% probability displacement ellipsoids (arbitrary spheres for H atoms).

## supplementary crystallographic information

### Comment

Epoxides are particularly versatile synthetic intermediates which can readily be converted into a wide range of polyfunctional compounds (Imashiro *et al.* 2004; Porter *et al.* 2000; Shing *et al.* 2006). A useful method for the synthesis of α, β-epoxy carbonyl compounds and related compounds is the Darzens condensation between a carbonyl compound and α-halo-carbonyl compound (Zhu *et al.* 1995). We report herein the crystal structure of the title compound.

The molecular structure of (I) is shown in Fig. 1. Bond lengths and angles in (I) are normal. The asymmetric unit of the title compound consists of two crystallographically independent molecules (Fig. 1) each of which adopts a *cis* configuration about the epoxides ring. The dihedral angle between the C1—C6 and C10—C15 ring is 82.14 (5)° and that between C16–21 and C25–30 phenyl ring is 84.15 (4)°. The dihedral angles between the epoxy ring and chlorophenyl rings are essentially identical for the two independent molecules [62.50 (9)° and 62.67 (9)°]. The crystal packing is stabilized by N—H···0 and C—H···0 hydrogen bonding (Table 1).

### Experimental

2-Chloro-*N*-phenylacetamide (0.17 g, 1.0 mmol) and potassium hydroxide (0.112 g, 2.0 mmol) were dissolved in acetonitrile (2 ml). To the solution was added 3-chlorophenylaldehyde (0.15 g, 1.0 mmol) at 298 K, the solution was stirred for 60 min and removal of solvent under reduced pressure, the residue was purified through column chromatography. Single crystals suitable for X-ray diffraction were obtained by slow evaporation of an ethyl acetate solution at room temperature for 2 d.

### Refinement

H atoms on N atoms were located in a difference Fourier map and refined isotropically, with restrains of N—H = 0.89±0.01 Å. Other H atoms were positioned geometrically with C—H = 0.93 and 0.98 Å, for aromatic and methine H atoms, respectively, and refined using a riding model, with *U*_iso_(H) =1.2*U*_eq_(C).

### Crystal data

**Table:**

C_15_H_12_ClNO_2_	*F*(000) = 568
*M**_r_* = 273.71	*D*_x_ = 1.409 Mg m^−^^3^
Monoclinic, *P*2_1_	Cu *K*α radiation, λ = 1.54184 Å
Hall symbol: P 2yb	Cell parameters from 12871 reflections
*a* = 5.4480 (1) Å	θ = 2.1–69.4°
*b* = 11.1481 (2) Å	µ = 2.60 mm^−^^1^
*c* = 21.3152 (4) Å	*T* = 295 K
β = 94.472 (2)°	Block, colorless
*V* = 1290.63 (4) Å^3^	0.40 × 0.30 × 0.30 mm
*Z* = 4	

### Data collection

**Table:**

Oxford Diffraction Gemini S Ultra diffractometer	4775 independent reflections
Radiation source: Enhance Ultra (Cu) X-ray Source	4453 reflections with *I* > 2σ(*I*)
mirror	*R*_int_ = 0.030
Detector resolution: 15.9149 pixels mm^-1^	θ_max_ = 69.7°, θ_min_ = 2.1°
ω scans	*h* = −6→5
Absorption correction: multi-scan (*CrysAlis PRO*; Oxford Diffraction, 2009)	*k* = −13→13
*T*_min_ = 0.423, *T*_max_ = 0.510	*l* = −25→25
19148 measured reflections	

### Refinement

**Table:**

Refinement on *F*^2^	Secondary atom site location: difference Fourier map
Least-squares matrix: full	Hydrogen site location: inferred from neighbouring sites
*R*[*F*^2^ > 2σ(*F*^2^)] = 0.028	H atoms treated by a mixture of independent and constrained refinement
*wR*(*F*^2^) = 0.072	*w* = 1/[σ^2^(*F*_o_^2^) + (0.044*P*)^2^ + 0.0893*P*] where *P* = (*F*_o_^2^ + 2*F*_c_^2^)/3
*S* = 1.00	(Δ/σ)_max_ = 0.001
4775 reflections	Δρ_max_ = 0.16 e Å^−^^3^
351 parameters	Δρ_min_ = −0.32 e Å^−^^3^
3 restraints	Absolute structure: Flack (1983), 2223 Friedel pairs
Primary atom site location: structure-invariant direct methods	Flack parameter: 0.000 (9)

### Special details

**Table:**

Geometry. All e.s.d.'s (except the e.s.d. in the dihedral angle between two l.s. planes) are estimated using the full covariance matrix. The cell e.s.d.'s are taken into account individually in the estimation of e.s.d.'s in distances, angles and torsion angles; correlations between e.s.d.'s in cell parameters are only used when they are defined by crystal symmetry. An approximate (isotropic) treatment of cell e.s.d.'s is used for estimating e.s.d.'s involving l.s. planes.
Refinement. Refinement of *F*^2^ against ALL reflections. The weighted *R*-factor *wR* and goodness of fit *S* are based on *F*^2^, conventional *R*-factors *R* are based on *F*, with *F* set to zero for negative *F*^2^. The threshold expression of *F*^2^ > σ(*F*^2^) is used only for calculating *R*-factors(gt) *etc*. and is not relevant to the choice of reflections for refinement. *R*-factors based on *F*^2^ are statistically about twice as large as those based on *F*, and *R*- factors based on ALL data will be even larger.

### Fractional atomic coordinates and isotropic or equivalent isotropic displacement parameters (Å^2^)

**Table:**

	*x*	*y*	*z*	*U*_iso_*/*U*_eq_	
Cl2	0.94031 (10)	0.56442 (6)	0.21597 (3)	0.07852 (18)	
Cl1	0.50893 (11)	0.28922 (5)	0.71602 (3)	0.07628 (16)	
N2	1.1469 (2)	0.78718 (12)	−0.07435 (6)	0.0417 (3)	
O4	0.7412 (2)	0.80333 (12)	−0.05625 (5)	0.0544 (3)	
O3	1.2753 (2)	0.95679 (10)	0.01702 (6)	0.0532 (3)	
O2	0.7705 (2)	0.71550 (11)	0.52655 (6)	0.0574 (3)	
O1	0.2463 (2)	0.56111 (12)	0.44875 (5)	0.0550 (3)	
N1	0.6527 (2)	0.55043 (12)	0.43108 (6)	0.0451 (3)	
C6	0.5906 (3)	0.47577 (15)	0.63907 (7)	0.0465 (4)	
H6	0.4543	0.5115	0.6550	0.056*	
C20	1.1985 (3)	0.78579 (15)	0.08902 (7)	0.0403 (3)	
C22	1.1318 (3)	0.90707 (14)	0.06459 (7)	0.0445 (3)	
H22	1.0876	0.9645	0.0967	0.053*	
C21	1.0566 (3)	0.73574 (15)	0.13417 (7)	0.0462 (4)	
H21	0.9217	0.7772	0.1474	0.055*	
C25	1.1310 (3)	0.69572 (13)	−0.12107 (6)	0.0364 (3)	
C13	0.6367 (3)	0.29390 (16)	0.28639 (7)	0.0467 (3)	
H13	0.6364	0.2383	0.2539	0.056*	
C10	0.6416 (3)	0.46247 (13)	0.38252 (7)	0.0384 (3)	
C24	0.9560 (3)	0.83310 (14)	−0.04600 (7)	0.0423 (3)	
C26	0.9306 (3)	0.68800 (14)	−0.16549 (6)	0.0406 (3)	
H26	0.8009	0.7420	−0.1645	0.049*	
C2	0.8708 (3)	0.30841 (17)	0.63764 (9)	0.0579 (4)	
H2	0.9211	0.2329	0.6521	0.069*	
C29	1.3159 (3)	0.52760 (15)	−0.16920 (8)	0.0473 (4)	
H29	1.4457	0.4737	−0.1706	0.057*	
C5	0.7174 (3)	0.53523 (15)	0.59396 (7)	0.0439 (3)	
C30	1.3245 (3)	0.61452 (14)	−0.12309 (7)	0.0430 (3)	
H30	1.4589	0.6189	−0.0935	0.052*	
C16	1.1176 (3)	0.62478 (16)	0.15900 (8)	0.0501 (4)	
C9	0.4602 (3)	0.59164 (14)	0.46030 (7)	0.0433 (3)	
C7	0.6352 (3)	0.65669 (15)	0.57293 (7)	0.0477 (4)	
H7	0.5868	0.7092	0.6067	0.057*	
C8	0.5170 (3)	0.68526 (15)	0.51004 (8)	0.0484 (4)	
H8	0.4025	0.7532	0.5088	0.058*	
C1	0.6677 (3)	0.36400 (16)	0.65992 (8)	0.0509 (4)	
C17	1.3165 (3)	0.56100 (17)	0.14013 (8)	0.0560 (4)	
H17	1.3554	0.4859	0.1571	0.067*	
C14	0.8318 (3)	0.29880 (17)	0.33223 (8)	0.0546 (4)	
H14	0.9620	0.2452	0.3306	0.066*	
C11	0.4450 (3)	0.45718 (14)	0.33707 (7)	0.0446 (4)	
H11	0.3143	0.5105	0.3385	0.053*	
C4	0.9242 (3)	0.48034 (18)	0.57174 (8)	0.0555 (4)	
H4	1.0134	0.5193	0.5423	0.067*	
C27	0.9252 (3)	0.59935 (16)	−0.21130 (7)	0.0461 (4)	
H27	0.7906	0.5940	−0.2408	0.055*	
C23	1.0199 (3)	0.93147 (14)	0.00091 (8)	0.0463 (4)	
H23	0.9122	1.0019	−0.0028	0.056*	
C19	1.3997 (3)	0.72225 (16)	0.07008 (7)	0.0485 (4)	
H19	1.4960	0.7548	0.0402	0.058*	
C3	0.9960 (4)	0.36815 (19)	0.59349 (9)	0.0620 (5)	
H3	1.1325	0.3319	0.5779	0.074*	
C18	1.4563 (3)	0.61121 (17)	0.09554 (8)	0.0569 (4)	
H18	1.5909	0.5693	0.0825	0.068*	
C28	1.1165 (3)	0.51936 (15)	−0.21357 (7)	0.0465 (4)	
H28	1.1122	0.4604	−0.2445	0.056*	
C15	0.8354 (3)	0.38212 (16)	0.38009 (8)	0.0484 (4)	
H15	0.9669	0.3845	0.4106	0.058*	
C12	0.4439 (3)	0.37255 (15)	0.28980 (7)	0.0465 (4)	
H12	0.3107	0.3687	0.2598	0.056*	
H1	0.804 (2)	0.578 (2)	0.4432 (9)	0.068 (6)*	
H16	1.299 (2)	0.8100 (17)	−0.0620 (8)	0.057 (5)*	

### Atomic displacement parameters (Å^2^)

**Table:**

	*U*^11^	*U*^22^	*U*^33^	*U*^12^	*U*^13^	*U*^23^
Cl2	0.0624 (3)	0.0986 (4)	0.0746 (3)	−0.0042 (3)	0.0055 (2)	0.0396 (3)
Cl1	0.0844 (4)	0.0729 (3)	0.0736 (3)	0.0089 (3)	0.0196 (3)	0.0186 (3)
N2	0.0410 (7)	0.0456 (6)	0.0381 (6)	−0.0039 (6)	0.0015 (5)	−0.0040 (5)
O4	0.0458 (7)	0.0632 (8)	0.0550 (7)	−0.0043 (6)	0.0089 (5)	−0.0090 (6)
O3	0.0640 (8)	0.0475 (6)	0.0487 (6)	−0.0142 (5)	0.0080 (5)	−0.0033 (5)
O2	0.0684 (8)	0.0541 (7)	0.0502 (6)	−0.0205 (6)	0.0078 (6)	−0.0091 (5)
O1	0.0466 (6)	0.0663 (8)	0.0527 (6)	−0.0062 (6)	0.0083 (5)	−0.0134 (6)
N1	0.0435 (7)	0.0504 (8)	0.0411 (6)	−0.0064 (6)	0.0025 (5)	−0.0109 (6)
C6	0.0432 (8)	0.0540 (9)	0.0422 (8)	0.0039 (7)	0.0029 (6)	−0.0088 (7)
C20	0.0404 (8)	0.0451 (7)	0.0347 (7)	−0.0003 (7)	−0.0013 (5)	−0.0062 (6)
C22	0.0544 (9)	0.0407 (8)	0.0387 (8)	−0.0025 (7)	0.0059 (7)	−0.0073 (6)
C21	0.0426 (9)	0.0527 (9)	0.0433 (8)	0.0036 (7)	0.0033 (6)	0.0012 (7)
C25	0.0375 (7)	0.0392 (7)	0.0327 (6)	−0.0048 (6)	0.0043 (5)	0.0008 (6)
C13	0.0534 (9)	0.0470 (8)	0.0408 (8)	−0.0052 (8)	0.0096 (6)	−0.0101 (7)
C10	0.0401 (8)	0.0411 (7)	0.0343 (7)	−0.0047 (6)	0.0047 (6)	−0.0038 (6)
C24	0.0503 (9)	0.0402 (7)	0.0365 (7)	−0.0025 (7)	0.0041 (6)	0.0023 (6)
C26	0.0413 (8)	0.0456 (8)	0.0347 (7)	0.0025 (6)	0.0010 (6)	0.0027 (6)
C2	0.0575 (10)	0.0558 (11)	0.0589 (10)	0.0108 (9)	−0.0044 (8)	−0.0112 (8)
C29	0.0414 (8)	0.0453 (8)	0.0560 (9)	0.0021 (7)	0.0097 (7)	−0.0029 (7)
C5	0.0422 (8)	0.0523 (9)	0.0367 (7)	−0.0031 (7)	−0.0004 (6)	−0.0134 (6)
C30	0.0356 (8)	0.0489 (8)	0.0443 (8)	−0.0037 (6)	0.0013 (6)	0.0005 (7)
C16	0.0461 (9)	0.0588 (10)	0.0441 (8)	−0.0067 (7)	−0.0045 (7)	0.0052 (7)
C9	0.0509 (9)	0.0428 (8)	0.0362 (7)	−0.0029 (7)	0.0031 (6)	−0.0031 (6)
C7	0.0532 (9)	0.0488 (8)	0.0415 (8)	−0.0052 (7)	0.0065 (7)	−0.0130 (7)
C8	0.0595 (10)	0.0426 (8)	0.0437 (8)	−0.0021 (7)	0.0071 (7)	−0.0070 (7)
C1	0.0498 (9)	0.0571 (10)	0.0452 (8)	−0.0007 (7)	0.0001 (7)	−0.0057 (7)
C17	0.0622 (10)	0.0495 (9)	0.0540 (9)	0.0070 (8)	−0.0099 (8)	0.0016 (8)
C14	0.0404 (8)	0.0599 (10)	0.0640 (10)	0.0072 (8)	0.0066 (7)	−0.0137 (9)
C11	0.0481 (9)	0.0469 (8)	0.0376 (8)	0.0080 (7)	−0.0030 (6)	−0.0010 (6)
C4	0.0454 (9)	0.0731 (11)	0.0487 (9)	−0.0022 (8)	0.0086 (7)	−0.0116 (8)
C27	0.0444 (9)	0.0590 (10)	0.0343 (7)	−0.0092 (7)	−0.0006 (6)	0.0003 (7)
C23	0.0557 (9)	0.0383 (7)	0.0451 (8)	0.0016 (7)	0.0057 (7)	−0.0007 (6)
C19	0.0438 (9)	0.0607 (10)	0.0413 (8)	0.0030 (7)	0.0042 (6)	−0.0066 (7)
C3	0.0488 (10)	0.0725 (12)	0.0648 (11)	0.0149 (9)	0.0050 (8)	−0.0172 (10)
C18	0.0538 (10)	0.0642 (11)	0.0520 (9)	0.0178 (8)	−0.0001 (8)	−0.0079 (8)
C28	0.0553 (10)	0.0442 (8)	0.0414 (8)	−0.0111 (7)	0.0119 (7)	−0.0069 (6)
C15	0.0358 (8)	0.0588 (10)	0.0500 (9)	−0.0003 (7)	−0.0013 (6)	−0.0076 (7)
C12	0.0525 (10)	0.0515 (9)	0.0344 (7)	0.0001 (7)	−0.0043 (6)	−0.0018 (6)

### Geometric parameters (Å, °)

**Table:**

Cl2—C16	1.7446 (18)	C26—H26	0.9300
Cl1—C1	1.7419 (19)	C2—C3	1.376 (3)
N2—C24	1.344 (2)	C2—C1	1.384 (2)
N2—C25	1.4231 (19)	C2—H2	0.9300
N2—H16	0.886 (9)	C29—C30	1.379 (2)
O4—C24	1.2198 (19)	C29—C28	1.387 (2)
O3—C23	1.435 (2)	C29—H29	0.9300
O3—C22	1.4388 (19)	C5—C4	1.397 (2)
O2—C7	1.437 (2)	C5—C7	1.484 (2)
O2—C8	1.439 (2)	C30—H30	0.9300
O1—C9	1.220 (2)	C16—C17	1.382 (3)
N1—C9	1.342 (2)	C9—C8	1.503 (2)
N1—C10	1.4237 (19)	C7—C8	1.476 (2)
N1—H1	0.898 (9)	C7—H7	0.9800
C6—C1	1.378 (2)	C8—H8	0.9800
C6—C5	1.394 (2)	C17—C18	1.382 (3)
C6—H6	0.9300	C17—H17	0.9300
C20—C19	1.391 (2)	C14—C15	1.379 (2)
C20—C21	1.397 (2)	C14—H14	0.9300
C20—C22	1.484 (2)	C11—C12	1.380 (2)
C22—C23	1.469 (2)	C11—H11	0.9300
C22—H22	0.9800	C4—C3	1.380 (3)
C21—C16	1.376 (2)	C4—H4	0.9300
C21—H21	0.9300	C27—C28	1.376 (3)
C25—C26	1.391 (2)	C27—H27	0.9300
C25—C30	1.393 (2)	C23—H23	0.9800
C13—C12	1.374 (2)	C19—C18	1.377 (3)
C13—C14	1.388 (2)	C19—H19	0.9300
C13—H13	0.9300	C3—H3	0.9300
C10—C11	1.388 (2)	C18—H18	0.9300
C10—C15	1.388 (2)	C28—H28	0.9300
C24—C23	1.506 (2)	C15—H15	0.9300
C26—C27	1.388 (2)	C12—H12	0.9300
			
C24—N2—C25	125.48 (13)	N1—C9—C8	116.11 (14)
C24—N2—H16	119.6 (12)	O2—C7—C8	59.18 (11)
C25—N2—H16	114.7 (12)	O2—C7—C5	117.74 (14)
C23—O3—C22	61.51 (10)	C8—C7—C5	124.83 (13)
C7—O2—C8	61.78 (11)	O2—C7—H7	114.5
C9—N1—C10	125.66 (13)	C8—C7—H7	114.5
C9—N1—H1	119.0 (14)	C5—C7—H7	114.5
C10—N1—H1	115.4 (14)	O2—C8—C7	59.04 (11)
C1—C6—C5	119.83 (15)	O2—C8—C9	118.44 (14)
C1—C6—H6	120.1	C7—C8—C9	122.83 (14)
C5—C6—H6	120.1	O2—C8—H8	115.0
C19—C20—C21	119.18 (15)	C7—C8—H8	115.0
C19—C20—C22	122.63 (14)	C9—C8—H8	115.0
C21—C20—C22	118.17 (14)	C6—C1—C2	121.74 (17)
O3—C22—C23	59.11 (10)	C6—C1—Cl1	119.94 (14)
O3—C22—C20	117.82 (13)	C2—C1—Cl1	118.31 (14)
C23—C22—C20	124.43 (13)	C16—C17—C18	118.58 (17)
O3—C22—H22	114.6	C16—C17—H17	120.7
C23—C22—H22	114.6	C18—C17—H17	120.7
C20—C22—H22	114.6	C15—C14—C13	120.88 (16)
C16—C21—C20	119.60 (16)	C15—C14—H14	119.6
C16—C21—H21	120.2	C13—C14—H14	119.6
C20—C21—H21	120.2	C12—C11—C10	119.85 (15)
C26—C25—C30	119.64 (13)	C12—C11—H11	120.1
C26—C25—N2	121.50 (13)	C10—C11—H11	120.1
C30—C25—N2	118.84 (13)	C3—C4—C5	119.86 (18)
C12—C13—C14	118.97 (15)	C3—C4—H4	120.1
C12—C13—H13	120.5	C5—C4—H4	120.1
C14—C13—H13	120.5	C28—C27—C26	120.77 (14)
C11—C10—C15	119.60 (14)	C28—C27—H27	119.6
C11—C10—N1	121.55 (14)	C26—C27—H27	119.6
C15—C10—N1	118.84 (13)	O3—C23—C22	59.38 (10)
O4—C24—N2	125.52 (14)	O3—C23—C24	118.15 (14)
O4—C24—C23	119.07 (15)	C22—C23—C24	122.42 (13)
N2—C24—C23	115.39 (14)	O3—C23—H23	115.1
C27—C26—C25	119.72 (14)	C22—C23—H23	115.1
C27—C26—H26	120.1	C24—C23—H23	115.1
C25—C26—H26	120.1	C18—C19—C20	120.05 (16)
C3—C2—C1	118.03 (17)	C18—C19—H19	120.0
C3—C2—H2	121.0	C20—C19—H19	120.0
C1—C2—H2	121.0	C2—C3—C4	121.72 (17)
C30—C29—C28	120.94 (15)	C2—C3—H3	119.1
C30—C29—H29	119.5	C4—C3—H3	119.1
C28—C29—H29	119.5	C19—C18—C17	121.11 (17)
C6—C5—C4	118.79 (16)	C19—C18—H18	119.4
C6—C5—C7	119.21 (14)	C17—C18—H18	119.4
C4—C5—C7	121.95 (16)	C27—C28—C29	119.25 (15)
C29—C30—C25	119.69 (14)	C27—C28—H28	120.4
C29—C30—H30	120.2	C29—C28—H28	120.4
C25—C30—H30	120.2	C14—C15—C10	119.71 (14)
C21—C16—C17	121.48 (17)	C14—C15—H15	120.1
C21—C16—Cl2	118.95 (14)	C10—C15—H15	120.1
C17—C16—Cl2	119.56 (13)	C13—C12—C11	120.99 (15)
O1—C9—N1	125.40 (14)	C13—C12—H12	119.5
O1—C9—C8	118.47 (14)	C11—C12—H12	119.5
			
C23—O3—C22—C20	115.43 (15)	O1—C9—C8—C7	106.3 (2)
C19—C20—C22—O3	3.3 (2)	N1—C9—C8—C7	−75.2 (2)
C21—C20—C22—O3	−178.56 (13)	C5—C6—C1—C2	0.5 (2)
C19—C20—C22—C23	73.3 (2)	C5—C6—C1—Cl1	−179.73 (12)
C21—C20—C22—C23	−108.56 (18)	C3—C2—C1—C6	0.0 (3)
C19—C20—C21—C16	−0.1 (2)	C3—C2—C1—Cl1	−179.76 (14)
C22—C20—C21—C16	−178.28 (14)	C21—C16—C17—C18	0.4 (2)
C24—N2—C25—C26	35.2 (2)	Cl2—C16—C17—C18	−179.17 (13)
C24—N2—C25—C30	−146.60 (14)	C12—C13—C14—C15	−0.9 (3)
C9—N1—C10—C11	36.7 (2)	C15—C10—C11—C12	−0.2 (2)
C9—N1—C10—C15	−144.72 (16)	N1—C10—C11—C12	178.34 (15)
C25—N2—C24—O4	−0.2 (2)	C6—C5—C4—C3	1.4 (2)
C25—N2—C24—C23	−178.62 (13)	C7—C5—C4—C3	178.88 (15)
C30—C25—C26—C27	0.1 (2)	C25—C26—C27—C28	−0.4 (2)
N2—C25—C26—C27	178.31 (14)	C22—O3—C23—C24	−112.95 (15)
C1—C6—C5—C4	−1.2 (2)	C20—C22—C23—O3	−104.43 (17)
C1—C6—C5—C7	−178.74 (14)	O3—C22—C23—C24	105.87 (17)
C28—C29—C30—C25	−0.3 (2)	C20—C22—C23—C24	1.4 (3)
C26—C25—C30—C29	0.2 (2)	O4—C24—C23—O3	173.79 (14)
N2—C25—C30—C29	−178.00 (14)	N2—C24—C23—O3	−7.7 (2)
C20—C21—C16—C17	−0.2 (2)	O4—C24—C23—C22	103.94 (19)
C20—C21—C16—Cl2	179.30 (12)	N2—C24—C23—C22	−77.5 (2)
C10—N1—C9—O1	−1.3 (3)	C21—C20—C19—C18	0.3 (2)
C10—N1—C9—C8	−179.71 (14)	C22—C20—C19—C18	178.39 (14)
C8—O2—C7—C5	115.96 (15)	C1—C2—C3—C4	0.2 (3)
C6—C5—C7—O2	179.81 (14)	C5—C4—C3—C2	−0.9 (3)
C4—C5—C7—O2	2.4 (2)	C20—C19—C18—C17	−0.2 (3)
C6—C5—C7—C8	−110.02 (19)	C16—C17—C18—C19	−0.2 (3)
C4—C5—C7—C8	72.5 (2)	C26—C27—C28—C29	0.4 (2)
C7—O2—C8—C9	−113.21 (16)	C30—C29—C28—C27	0.0 (2)
C5—C7—C8—O2	−104.19 (18)	C13—C14—C15—C10	−0.1 (3)
O2—C7—C8—C9	105.89 (18)	C11—C10—C15—C14	0.7 (2)
C5—C7—C8—C9	1.7 (3)	N1—C10—C15—C14	−177.90 (16)
O1—C9—C8—O2	175.98 (14)	C14—C13—C12—C11	1.4 (3)
N1—C9—C8—O2	−5.5 (2)	C10—C11—C12—C13	−0.9 (3)

### Hydrogen-bond geometry (Å, °)

**Table:**

*D*—H···*A*	*D*—H	H···*A*	*D*···*A*	*D*—H···*A*
N1—H1···O1^i^	0.90 (1)	2.41 (1)	3.2287 (15)	152
N2—H16···O4^i^	0.89 (1)	2.40 (1)	3.2357 (16)	156
C4—H4···O1^i^	0.93	2.49	3.388 (2)	162
C15—H15···O1^i^	0.93	2.58	3.259 (2)	130
C19—H19···O4^i^	0.93	2.59	3.5087 (19)	168

Symmetry codes: (i) *x*+1, *y*, *z*.
